# Composite Multifocal Basal Cell carcinoma and Precursor B Acute Lymphoblastic Leukemia: Case report

**DOI:** 10.1186/1746-1596-2-32

**Published:** 2007-08-23

**Authors:** Dina El Demellawy, Monalisa Sur, Catherine Ross, Franco DeNardi, Salem Alowami

**Affiliations:** 1 Northern Ontario School Of Medicine, Department Of Pathology and Laboratory Medicine, West Campus, Thunder Bay, Ontario, Canada; 2 McMaster University, Department Of Pathology and Laboratory Medicine, Hamilton, Ontario, Canada

## Abstract

Synchronous composite tumors though described are uncommon. Moreover, simultaneous occurrence of synchronous tumors involving the same tissue or organ at multiple sites is even less common. We report a case of acute lymphoblastic leukemia (ALL) and basal cell carcinoma (BCC) occurring simultaneously in multiple skin sites. Several cases showing an association between cutaneous malignancies and lymphoproliferative disorders have been reported. Some of these cases included ALL and BCC and occurred often in the pediatric population with the BCC arising as a post-ALL therapy sequela. Other rare genetic causes may be considered. To our knowledge this is the first time that the synchronous occurrence of these two malignant processes in the same tissue involving multiple sites in an elderly patient is described.

## Background

Composite malignancies are an interesting phenomenon that gives rise to questions related to etiologies, diagnostic implications, and treatment. We present an interesting case of a composite multifocal acute lymphoblastic leukemia (ALL) and basal cell carcinoma (BCC). Independently the two malignancies occur in two opposite extremes of age and their occurrence as a composite tumor is extremely unusual. In fact, to the best of our knowledge, composite ALL and BCC has never been reported before in the English literature.

## Case presentation

### Clinical summary

A previously healthy 76 year-old caucasian man presented with multiple slowly growing translucent tan and pink skin patches and nodules, involving the left upper limb, right supraclavicular fossa and the posterior surface of the right ear. These lesions showed ill-defined borders and were of variable sizes, which ranged from 1 cm to 3.5 cm in maximum dimension. Skin ulceration, lymphadenopathy and organomegaly were absent. Clinically, the skin lesions were diagnosed as BCC and were treated by wide excision.

### Pathological findings

**Figure 1:**
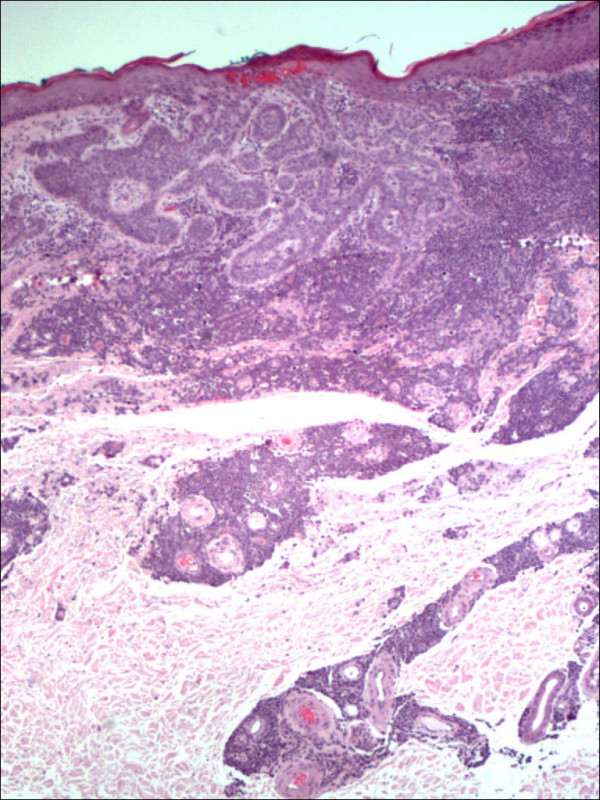
Dermal infiltration by basal cell carcinoma and lymphoblastic lymphoma. The latter extends to involve the deeper dermis with peri-vascular accentuation (HE 40×).

**Figure 2:**
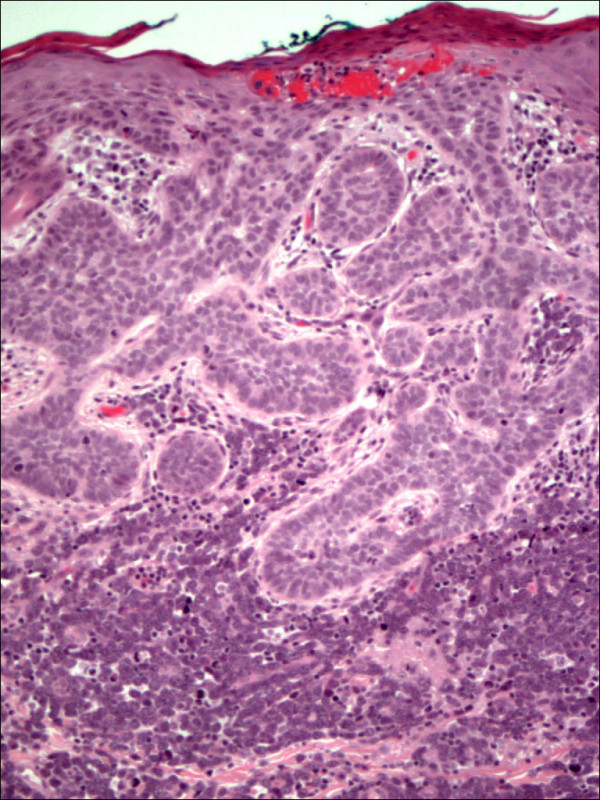
Skin with basal cell carcinoma showing continuity with the overlying epidermis and intervening dermis infiltrated by large blasts of acute lymphoblastic lymphoma (HE 100×).

**Figure 3:**
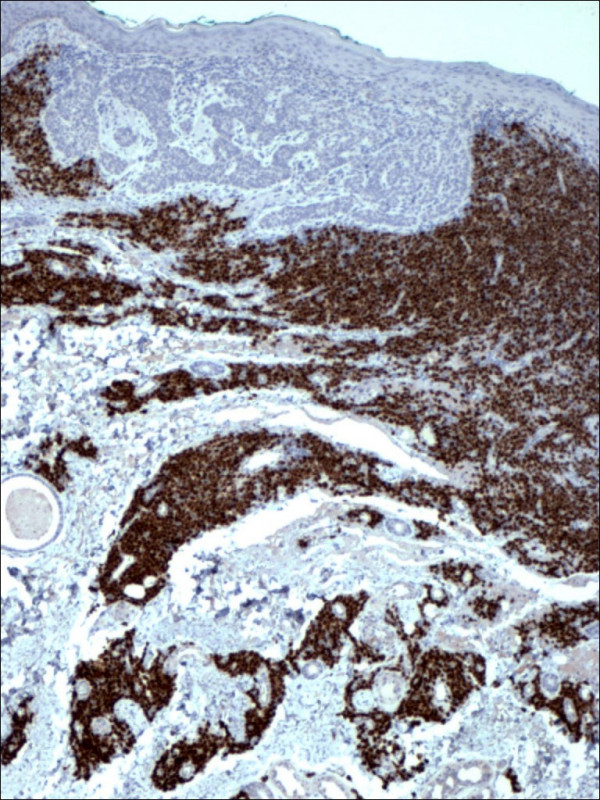
Restricted expression of TdT within the lymphocytes in lymphoblastic lymphoma in contrast to basal cell carcinoma cells (TdT 100×).

**Figure 4:**
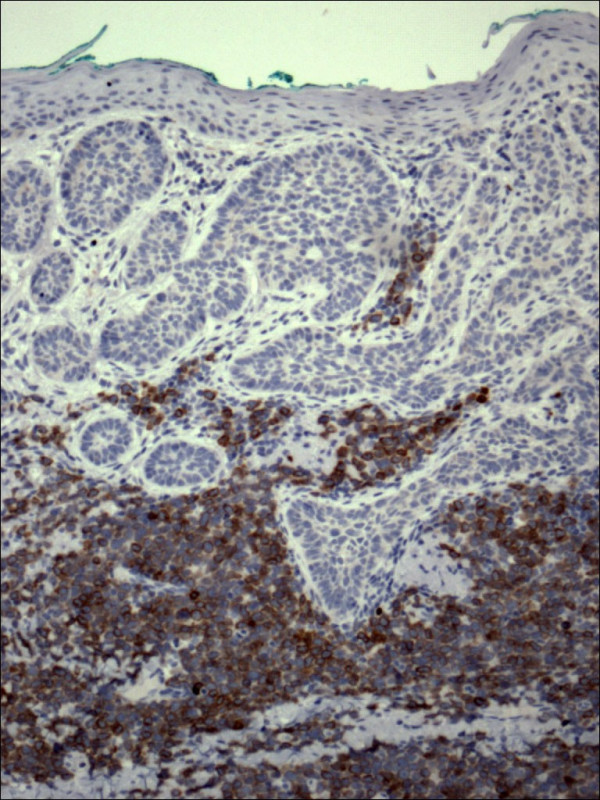
Restricted expression of CD79a within the lymphocytes in lymphoblastic leukemia in contrast to basal cell carcinoma (CD79a 100×).

**Figure 5:**
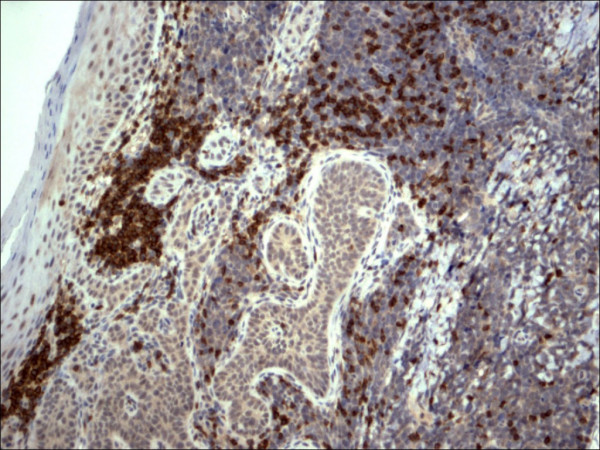
Expression of CD3 within reactive mature looking T lymphocytes and absence of staining in both lymphoblastic lymphoma and basal cell carcinoma (CD3 100×).

**Figure 6:**
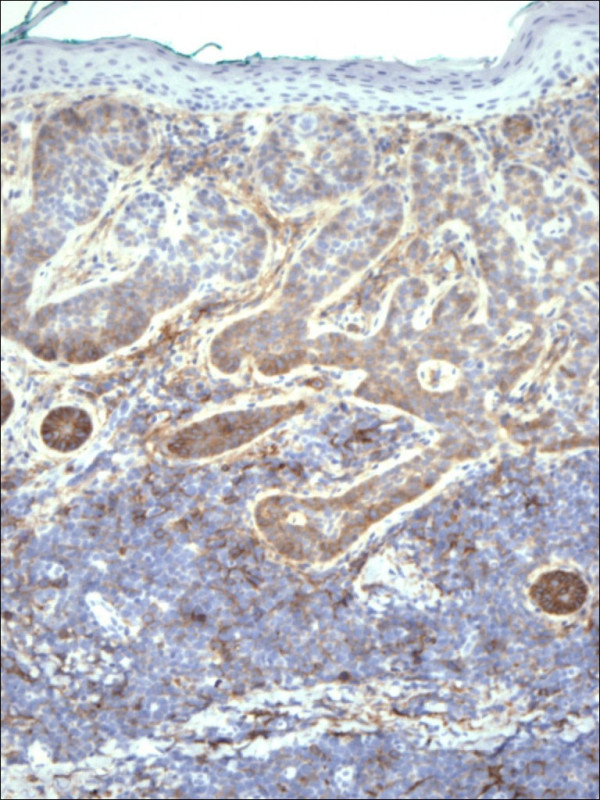
Simultaneous expression of CD10 within the lymphocytes in lymphoblastic lymphoma and basal cell carcinoma cells (CD10 400×).

**Figure 7:**
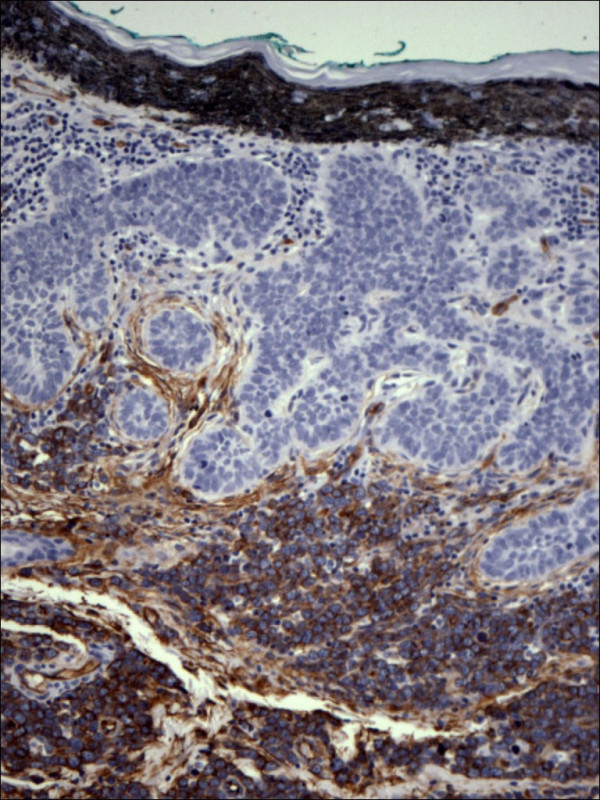
Expression of CD34 within lymphocytes in lymphoblastic leukemia, native epidermis and dermal vessels and absence of staining in basal cell carcinoma (CD34 100×).

**Figure 8:**
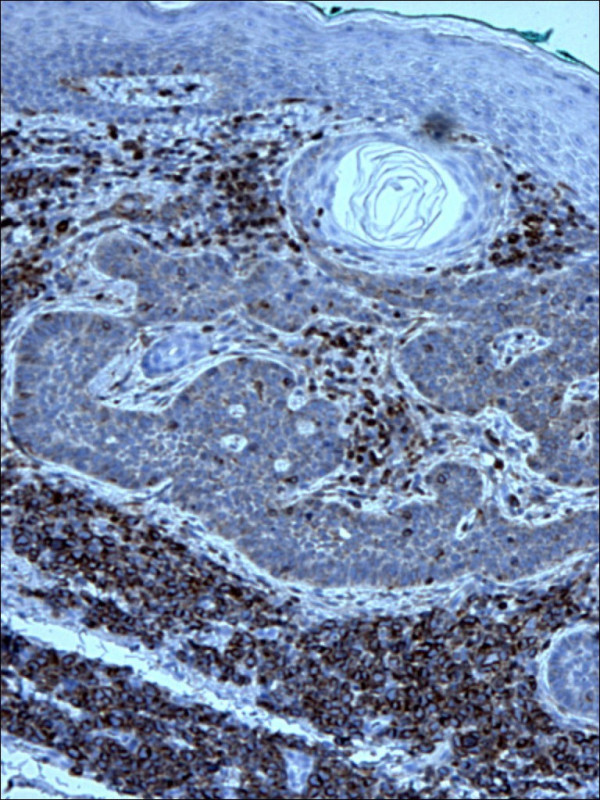
Restricted expression of Bcl-2 within the lymphocytes in lymphoblastic leukemia and reactive T lymphocytes in contrast to basal cell carcinoma cells (Bcl-2 100×)

Pathology confirmed the presence of multiple BCCs and in addition the three biopsy sites showed diffuse infiltration of the dermis by large, atypical cells with blastic morphology and displaying perineural and perivascular accentuation (Fig. 1, 2). Occasional tumor vascular emboli were noted (Fig. 3). In addition, dense infiltrates of small mature lymphocytes were noted inbetween the invasive solid masses of BCC. A wide panel of immunohistochemistry was performed (Table 1 and fig. 3, 4, 5, 6, 7, 8) and accordingly the case was diagnosed as composite multifocal BCC and precursor B acute-ALL. Consequently, the patient was fully investigated. The peripheral blood showed a leukoerythroblastic picture. The bone marrow revealed pancytopenia and acute leukemia with a blast count greater than 20%. Flow cytometry results confirmed the diagnosis of precursor B-ALL. The patient received induction chemotherapy with high dose cyclophosphamide under mesna cover, doxorubicin, vincristine and high dose dexamethasone. A month and a half later he was readmitted and received methotrexate with leucovorin rescue and high dose cytosine arabinoside. Follow up for 2 years shows the patient to be still in remission.

**Table 1:** Immunohistochemical profiles of the two composite tumors

Marker	Basal cell carcinoma	Mature looking lymphocytes	Precursor B cell acute lymphoblastic leukemia
BER EP4	Diffusely Positive	Negative	Negative
TdT	Negative	Negative	Diffusely Positive
CD 10	Diffusely Positive	Negative	Diffusely Positive
CD 43	Negative	Diffusely Positive	Diffusely Positive
CD 79a	Negative	Negative	Diffusely Positive
Bcl-2	Negative	Diffusely Positive	Diffusely Positive
LCA (CD 45)	Negative	Diffusely Positive	Diffusely Positive
CD 34	Negative	Negative	Diffusely Positive
Myeloperioxidase	Negative	Negative	Negative
CD 117	Negative	Negative	Weakly Positive (<10%)
CD 99	Negative	Negative	Weakly Positive (<10%)
CD 3	Negative	Diffusely Positive	Negative
CD 5	Negative	Diffusely Positive	Negative
CD 7	Negative	Diffusely Positive	Negative
Ki 67 index	5%	0%	30–40%

## Discussion

Composite double malignancies involving similar and multiple locations are extremely rare. Such tumors usually occur in a setting of immune deficiency or a genetic predisposition. BCC and ALL are two tumors that affect different age groups with the former almost always occurring in elderly and the latter in children. ALL associated with metachronous but not synchronous BCC has been reported [1-3]. Such cases occurred in children who received radiotherapy as part of the treatment protocol of ALL and as a possible consequence of BCC. Similar cases of BCC have been reported after radiotherapy for brain tumors [4]. It is not surprising that such cases occurred in children, as 88% of patients with ALL are under 35 years of age with a median age of 20 years [5]. On the other hand, BCC are extremely rare in children with 45 case reports of BCCs in children aged 15 years or younger [6]. The occurrence of BCC is possibly a result of an ionizing "radiation induced" complication. The other but less likely etiological factor underlying the occurrence of the two tumors is ionizing radiation associated BCC in patients with nevoid BCC syndrome (NBCCS) or a sporadic BCC. NBCCS is an autosomal dominant disorder with a high degree of penetrance and variable expressivity, and is characterized by BCC, odontogenic keratocysts, palmar and/or plantar pits, and ectopic calcifications of the falx cerebri. More than 100 minor criteria have been described, but 2 major and 1 minor criterion or 1 major and 3 minor criteria are necessary for the diagnosis. The median interval between ionizing radiation induced BCC and X-ray exposure is greater than 20 years in older patients, however this interval may be as short as 3–5 years in younger patients [7]. To the best of our knowledge, composite multifocal ALL and BCC has never been reported before in the English literature. In the present case, the absence of any prior history of radiotherapy or immune deficiency and the mere occurrence in a previously healthy adult suggest a coincidental but unusual occurrence of composite tumors.

In our case, the presence of a dermal vasocentric undifferentiated large cell tumor in a setting of BCC occurring in an adult raised the differential of several hematological malignancies such as granulocytic sarcoma, acute myeloid leukemia, blastoid mantle cell lymphoma, ALL, and Burkitt's lymphoma, though the latter two tumors show a predilection for young age. In addition solid tumors such as neuroblastoma, malignant melanoma and Merkel cell carcinoma were also included. As the tumor was not in bone marrow and involved an elderly patient without a previous history of chemo- or radiotherapy, there was no consideration of haematogones in the differential diagnosis. With the aid of immunohistochemistry, the tumor was diagnosed as precursor B-ALL. Although skin involvement with B-ALL is frequent, it is interesting that this patient presented initially with a skin manifestation with multifocal BCC rather than the usual manifestations of ALL such as bone marrow failure, hepatosplenomegaly, or lymphadenopathy. The findings of pancytopenia and marrow failure were discovered only later.

In conclusion we report an unusual case of composite multifocal precursor B-ALL and BCC. To the best of our knowledge this is the first case report in the English literature of the synchronous multifocal occurrence of these tumors in an adult. The case presented here emphasizes the value of awareness of composite tumors. The awareness of occurrence of composite tumors is crucial so that the patient can receive adequate therapy. Consideration of tumors in the differential diagnosis that fit the morphology but not the usual demographic profile, even in the absence of clinical suspicion, is a safe practice if accompanied by further ancillary studies to document or rule them out.
